# Correlates of influenza vaccination among underserved Latinx middle-aged and older adults: a cross-sectional survey

**DOI:** 10.1186/s12889-022-13121-z

**Published:** 2022-05-06

**Authors:** Mohsen Bazargan, Adrienne Martinez-Hollingsworth, Sharon Cobb, Lucy W. Kibe

**Affiliations:** 1 Charles R Drew University of Medicine and Science (CDU), 1731 East 120th Street, Los Angeles, CA 90059 USA; 2 University of California, Los Angeles, 1100 Glendon Ave. Suite 900, Los Angeles, CA USA

**Keywords:** Influenza vaccination, Provider recommendation, Latinx, Hispanic, Older adults

## Abstract

**Background:**

Vaccination is a powerful tool in the fight against seasonal influenza, among underserved, middle-age and older, Latinx adults. Yet, vaccine hesitancy and inconsistent uptake in this population continues to represent a substantial challenge to public health. A better understanding of factors impacting influenza vaccination behaviors in this group could result in more effective messaging and initiatives promoting universal vaccination among Latinx.

**Methods:**

In this cross-sectional survey, we explore correlates of influenza vaccination uptake among underserved, Latinx, older adults. Our focus was on the role of socio-demographics, living arrangements, financial strain, access and satisfaction with medical care, and the presence of major chronic conditions in terms of vaccine uptake. Middle-aged and older Latinx residents diagnosed with diabetes and/or hypertension (*n*=165), were recruited from the South Los Angeles Service Plan Area (SPA), a historically under-resourced community. Bi-variate and multi-variate logistical regression were performed on survey data to explore independent correlates of influenza vaccination.

**Results:**

Almost half of underserved Latinx older adults in our study (45%) reported influenza vaccination within the 12 months prior to the study. The majority (~85%) reported receiving this recommendation from their primary care provider. However, thirty percent (30%) of those receiving this advice did not get the vaccine. A decreased likelihood of vaccination was significantly associated with living alone (*p*-value=0.026), lacking Medicare coverage (0.028), or higher levels of financial strain (0.020). Difficulty accessing medical care (*p*-value=0.008) or dissatisfaction with these experiences (*p*-value=0.001) were also strongly associated with decreased likelihood of vaccination. Participants diagnosed with COPD had 9.5 (CI: 1.76 – 51.3) higher odds of being vaccinated compared to those without; no correlation was detected for other chronic conditions.

**Conclusion:**

The high number of unvaccinated Latinx participants receiving a vaccine recommendation from a provider is consistent with studies among other ethnic/racial minority older adults and highlights the pivotal role of the provider in influenza vaccine adoption. Additional findings reflect negative impact of Social Determinates of Health on preventive care efforts in this group. Further efforts to quantify these associations are needed to explore structural and human factors impacting influenza vaccine uptake.

## Background

Underserved, older, Latinx populations demonstrate a consistent pattern of higher rates of infection, hospitalization and death from SARS-CoV-2 infection across multiple geographic areas compared to their White, non-Latinx counterparts [1–6]. Since its initial roll-out in the United States (US) in December 2020, public health authorities have lauded the COVID-19 vaccine as the “weapon that will win the war,” and have stressed vaccine acceptance and dissemination among this and other vulnerable populations [7]. COVID-19-positive patients who had not received the influenza vaccination within the last year had substantially greater odds of hospitalization and ICU admission when compared with those who were vaccinated [8]. In addition, association between previous influenza vaccination and reduced COVID-19 infection have been well-documented [9]. Furthermore, co‐infection of influenza and COVID-19 events will make diagnosis of either entity more difficult and could potentially increase morbidity [10]. Indeed, recent national data revealed that intent to vaccinate for COVID-19 is lower among those who have not being vaccinated against influenza during the 2019–2020 influenza season [11]. Prior literature on racial disparities in vaccinations have suggested potential sources of vaccine hesitancy that may retard this effort. In particular, current knowledge on the barriers of seasonal influenza vaccination rates among underserved, middle-age and older Latinx adults may provide opportunities to mitigate the status quo. The timely and useful insights will support the effort to increase wide-spread COVID-19 vaccine uptake in the Latinx community, currently most affected by the novel coronavirus. Preliminary 2019-2020 US projections indicated that the influenza season wrapped in the COVID-19 pandemic, with potential co-infections, would escalate the disease burden. With an estimated at 38 million influenza cases, ~400k hospitalizations, and ~22k deaths, the health system would be overwhelmed and stretched to the breaking point [12].

Despite having higher rates of disease, Latinx adults have lower rates of influenza vaccination compared to their White, non-Latinx counterparts [13–15]. Data on the last two influenza seasons (2018-19, 19-20) from the US Centers of Disease Control and Prevention (CDC) indicate vaccination rates among US Latinx remain below the target level set by Healthy People 2030, with less than four in ten US Latinx adults (37-38%) receiving the vaccine compared to over half (49-53%) of White, non-Latinx US adults [16]. Rates of influenza vaccination among US Latinx children (6 months- 17 years) were almost double (66-67%) that of US Latinx adults in the same period, and in fact, race and age remain the most consistent sociodemographic predictors of influenza vaccine [17, 18]. These variations by age indicate a need for targeted explorations of unique barriers facing middle-age and older US Latinx individuals who are at higher risk for both influenza and COVID-19.

Disparities in influenza vaccination among older US Latinx represent both a major challenge facing public health and reflect discrimination and persistent, systematic disenfranchisement within existing US healthcare structures [19]. Despite the heterogeneity of US Latinx groups, a legacy of unequal access to basic services and preventive care is shared across diverse Latinx communities [20–24]. Barriers to vaccine acceptance such as lack of trust and insufficient patient education are also connected to low acceptance among other ethnic/racial minorities, but may be compounded by additional, culturally informed barriers among US Latinx [17, 18, 25, 26]. Certain social and cultural norms characteristic of US Latinx groups, such as resource sharing in times of financial shortage and cultural concepts regarding care for the sick, similarly bear further investigation for their known contribution to influenza and COVID-19 prevention strategies and potential for transmission [27].

Explorations that provide targeted insight on how to stem the spread of influenza in the high-risk, aging US Latinx population may shed light on best practices to manage the COVID-19 pandemic that continues to rage out of control across this population. However, only a limited research has focused on influenza vaccination among Latinx adults [14, 25, 26, 28–34]. Additional exploratory studies are needed to shape effective interventional strategies to improve uptake in both the short and long-term among the underserved Latinx population. Therefore, the purpose of this study was to investigate seasonal influenza vaccination rates among underserved middle-age and older Latinx adults residing in South Los Angeles (South LA). Specifically, we aimed to examine correlates associated with influenza vaccination, with a particular focus on access and satisfaction with medical care. This is important as the target population includes underserved older Latino adults living in a critically under-resourced service area of Los Angeles County.

## Methods

### Recruitment

This cross-sectional survey occurred between 2018 and 2020**;** data collection was completed before the emergence of the SARS-CoV-2 virus (February 2020). Middle-aged and older Latinx adults residing in South Los Angeles (LA) (*n*=165) were recruited from fourteen senior centers and senior housing centers in the South Los Angeles Service Plan Area (SPA) 6. SPA 6 encompasses the communities of Athens, Compton, Crenshaw, Florence, Hyde Park, Lynwood, Paramount, and Watts, and is home to over one million people. Residents of SPA 6 disproportionately shoulder vaccination disparities compared to their counterparts across other LA County SPAs. Age adjusted mortality rate of pneumonia/influenza (per 100,000 population) is significantly higher in this service area compared to the rest of LA County and nationally (31.1 for South LA versus 22.5 population LA County and 15.9 for USA population) [35]. Such disparities are conceptually tied to the objectives of this project in that the design of this study recognizes a history and legacy of health inequity persistent in this geographic area.

### Inclusion and exclusion criteria

Self-identified Latinx residents, 55 years or older with a diagnosis of Type 2 diabetes and/or hypertension (HTN) were recruited. Participants must have been residents of SPA 6 for a minimum of 1 year. Individuals were excluded if they did not self-identify as Latinx, were outside of the requested age range, and were institutionalized or exhibiting cognitive impairment that required any prescribed medication be administered by a care giver.

### Data collection

Face-to-face interviews were conducted with participants by members of the study team to obtain demographic data, and information on socioeconomic status, health status and behaviors, care access and utilization.

### Data sharing

The datasets used and/or analyzed during the current study are available from the corresponding author on reasonable request.

### Measurements

#### Influenza vaccination

Participants were asked if they had ever had an influenza shot; if they responded yes, they were asked: “How long has it been since you had a shot to prevent the flu?” and “In the past 12 months, has a doctor recommended to you that you should have a flu shot?”

#### Sociodemographic covariates

Participants were asked their age, level of educational attainment, and living arrangements. Educational attainment was operationalized as a continuous variable (number of years for school attendance), with higher scores indicating more years of education.

#### Major chronic conditions and Chronic Obstructive Pulmonary Disease (COPD)

Participants were asked about their chronic illnesses, including cardiovascular conditions, such as high blood pressure, and diabetes mellitus (Type 2 diabetes), as well as respiratory conditions such as COPD, asthma, and bronchitis.

#### Living arrangement

Participants were asked about their living situation; these data were categorically grouped as either “living alone” or “living with at least one other individual”.

#### Financial strain

Five items were used to measure financial strain:

In the past 12 months, 1) How frequently were you unable to buy the amount of food your family should have? 2) How frequently were you unable to buy the clothes you feel your family should have? 3) How often were you unable to pay your rent or mortgage? 4) How frequently were you unable to pay your monthly bills? and 5) How often were you unable to make ends meet?

Items were on a Likert scale ranging from 1 (never) to 5 (always). A total “financial difficulty” score was calculated using the mean across five items. A high score was indicative of higher financial difficulty. These items are consistent with Pearlin’s list of chronic financial difficulties of low SES individuals [36] (Cronbach alpha = 0.92).

#### Access and satisfaction with medical care

A 7-item questionnaire assessed level of satisfaction and difficulty with access to, availability of and quality of medical care. Items were on a Likert scale ranging from 1 (low) to 5 (high). Principal component analysis was used to identify potential factors underlying the 7-item instrument that measures the access and satisfaction with medical care. Varimax rotation produced 2 distinct factors, explaining almost 61% of the variance. The first factor explained 33% of the variance, while the second factor explained 28% of the variance. All items had primary loadings exceeding 0.5, and none of the items had a cross-loading of greater than 0.4. The Cronbach α coefficient for these 4 items was calculated to be 0.81. A higher score on this index reflects a higher level of dissatisfaction and difficulty.

The first factor was associated with the 4 items that measure perceived difficulty to access to medical care. This factor is associated with the following items:

1. Overall, how difficult is it for you to get medical care?
2. How difficult would it be for you to get a routine physical exam if you wanted one?
3. How difficult is it for you to visit a doctor when you need medical care?
4. In the past 12 months, how frequently were you unable to visit a doctor because payment was necessary?
  The second factor produced by the Varimax rotation is associated with 3 items that measured satisfaction with medical care. This factor is associated with the following items:
5. Overall, how satisfied are you with your access to medical services?
6. Overall, how satisfied are you with how available medical care is for you?
7. Overall, how satisfied are you with your access to preventive services?

The Cronbach α coefficient for these 4 items was calculated to be 0.72. A higher score on this factor reflects a higher level of difficulty to access medical care.

### Data analysis

Descriptive statistics are reported for all variables measured. Means and standard deviations (SD) were used to summarize continuous variables; frequencies and percentages summarize categorical variables. In addition, at bivariate level, correlates of uptake of flu vaccination within last 12 months were examined using independent samples t-tests for continuous measures and chi-square tests for categorical measures, where appropriate. Independent variables used to compare participants who had flu vaccination within last 12 months with those who did not included 1) socio-demographic status (gender, age, education, financial strains, living arrangements, and health care coverage); 2) access, and satisfaction with medical care; 3) major chronic conditions; and 4) recommendation for flu vaccination by health care providers. Finally, at multivariate analysis, binary logistic regression technique was employed to document independent correlates of flu vaccination within last 12 months. A *p*-value of less than 0.05 was considered significant.

## Results

Our study sample characteristics are described in Table 1. Self-described Latinx individuals (*n*=165) between the ages of 55 and 95 years (mean= 70.4 ± 8.25) were included. Under one-third (29%) were 75 years or older, over two-thirds (69%) were women. One-third (33%) lived alone. Educational attainment was low, three-fourths (75%) had not attained a high school diploma. A low percentage of our participants described their health as “excellent” (8%), while the majority characterized their health as fair or poor (49%). Additional health conditions and chronic illnesses were noted: Type 2 diabetes (51%), HTN (79%), heart-related conditions (19%), and COPD (17%) (Table 1).

**Table 1:** Characteristics of participants (*n* = 165)

	n	%
***Gender***		
Male	51	30.9
Female	114	69.1
***Age***		
55-64	38	23.1
≥ 65	123	76.9
***Education***		
No high school diploma	124	75.2
High school diploma	41	24.8
***Living Alone***		
No	110	66.7
Yes	55	33.3
***Medicare Coverage***		
No	68	42.2
Yes	93	57.8
***COPD***		
No	129	83.2
Yes	26	16.8
***Heart Conditions***		
No	126	81.3
Yes	29	18.7
***HTN***		
No	33	21.3
Yes	122	78.7
***Diabetes***		
No	79	51.0
Yes	76	49.0
		Mean ± SD
***Age**** (years: 55 - 95)*		*70.4 ± 8.26*
***Education Attainment**** (1 – 16)*		*7.8 ± 4.27*
***Financial Strains**** (1: – 5)*		*3.5 ± 1.46*

### Flu vaccination

Almost half of our participants (45%, *n*=73) had been vaccinated within the 12 months prior to interview. Almost all (85%, *n*=139) reported their health care provider had told them that “they should have a flu shot” in the past 12 months; however, of those who received this recommendation, nearly one-third (30%, *n*=42) had not obtained a vaccine in the past 12 months.

### Bi-variate analysis

Table 2 presents the bivariate associations between the flu vaccination and other relevant variables. The following factors were noted to be associated with being less likely to have the flu vaccination within the past 12 months: living alone (*p*-value < 0.05), not being covered by Medicare (*p*-value < 0.05), higher level of financial strain (*p*-value < 0.05), higher level of difficulty accessing medical care (*p*-value < 0.01), a higher level of dissatisfaction with the medical care (*p*-value < 0.01), not receiving a flu vaccine recommendation from their health care provider (*p*-value < 0.001), not being diagnosed with COPD (*p*-value < .01) and HTN (*p*-value < 0.05), No association between other major chronic conditions and flu vaccination was detected.

**Table 2:** Bi-Variates Correlates of Flu Vaccination within Last 12 months (*n*= 165)

**Independent Variables**	**Flu Vaccination (Last 12 Months)**		Sig.
	No N (%) or (*X* ± SD)	Yes N (%) or (*X* ± SD)	
**Gender**			
Male	17 (33)	34 (67)	0.686
Female	41 (37)	71 (63)	
**Age**	(69.3 ± 8.79)	(71.0± 7.98)	0.517
**Education (Scale:** 1 – 16)	(7.9 ± 4.23)	(7.73 ± 4.30)	0.816
**Financial Strain** (scale: 1-5)	(3.15 ± 1.55)	(3.72 ± 1.38)	0.020
**Living Alone**			
No	32 (30)	76 (70)	0.026
Yes	27 (47)	29 (53)	
**Medicare Coverage**			
Yes	26 (46)	67 (64)	0.028
No	30 (45)	37 (55)	
**Flu Vaccination Recommended**			
No	16 (67)	8 (33)	0.001
Yes	42 (30)	96 (70)	
**Difficulty Accessing Medical Care**	(0.28 ± 1.33)	(-0.15 ± 0.73)	0.008
**Dissatisfaction with Medical Care**	(0.37 ± 0.94)	(-1.96 ± 0.98)	0.001
**COPD**			
No	51 (40)	77 (60)	0.002
Yes	2 (08)	24 (92)	
**HTN**			
No	12 (37)	20 (63)	0.026
Yes	41 (34)	81 (66)	
**Type 2 Diabetes**			
No	28 (36)	76 (64)	0.695
Yes	25 (33)	29 (67)	
**Heart condition**			
No	41 (33)	84 (67)	0.381
Yes	12 (41)	17 (59)	

### Multivariate logistic regression

The results of the binary logistic regression examining the correlation between flu vaccination and the independent variables are shown in Table 3. Participants who lived alone were 0.42 (CI: 0.18 – 0.99) times less likely to be vaccinated. In addition, participants with a higher level of difficulty accessing medical care had 0.58 (CI: 0.36 – 0.94) lower odds of being vaccinated. Similarly, participants with a higher level of dissatisfaction with medical care had 0.56 (CI: 0.35 – 0.91) lower odds of being vaccinated. As expected, participants who indicated that their provider had advised them to obtain a flu vaccination were 4.96 (CI: 1.45 – 16.9) times more likely to be vaccinated. After controlling for other relevant variables, no correlation was detected between being diagnosed with diabetes, hypertension, and heart conditions. However, adjusting for other variables, participants diagnosed with COPD had 9.5 (CI: 1.76 – 51.3) higher odds of being vaccinated compared with participants with no COPD conditions.

**Table 3:** Multivariate logistic regression model estimating correlates of Flu Vaccination among underserved middle and older latino adults (*N* = 165)

**Independent Variable**	**OR**	**95% OR**	**Sig.**
**Age**	1.021	.962 - 1.084	.488
Gender			
Male	1.000		
Female	1.417	0.556 - 3.611	0.465
**Education Attainment**	0.982	0.891 - 1.083	0.722
**live Alone**			
No	1.000		
Yes	**0.415**	**0.175 - 0.987**	**0.047**
**Medicare**			
No	1.000		
Yes	1.973	0.789 - 4.935	0.146
**Financial Strains**	1.060	0.752 - 1.495	0.740
**Provider Recommended Flu Shot**			
No	1.000		
Yes	**4.958**	**1.451 - 16.94**	**0.011**
**Access to Care**	**0.576**	**0.355 - 0.935**	**0.026**
**Satisfaction with Care**	**0.560**	**0.346 - 0.906**	**0.018**
**COPD**			
No	**1.000**		
Yes	**9.488**	**1.755 - 51.29**	**0.009**
**Diabetes**			
No	1.000		
Yes	1.562	0.635 - 3.842	0.332
**Heart Condition**			
No	1.000		
Yes	0.498	0.163 - 1.517	0.220
**HBP**			
No	1.000		
Yes	1.755	0.610 - 5.055	0.297
**-2 Log likelihood**	**147.926**		
**Nagelkerke R-Square**	**0.377**		

## Discussion

This study revealed that less than half (45%) of middle-age and older Latinx adult residents of South Los Angeles in our sample reported influenza vaccination within the 12 months prior to the interview. Almost 85% of our participants reported receiving vaccination recommendations from a healthcare provider, however, more than 30% of these participants did not adhere to their providers’ recommendations**.**A systematic review of recent studies show that one of the major determinants of influenza vaccination among older adults is recommendation by their healthcare provider [37]. Indeed, there are strong evidence that providers may have a profound influence on individuals’ intentions to receive vaccinations thus physicians have a unique role in promoting influenza vaccination rates at a national level [38]. Along those lines, it has consistently been documented that vaccination recommendation by primary care providers is an influential factor for immunization among older adults [39]. Knowing that almost one out of three participants did not adhere to their providers’ recommendations, it is necessary that providers of older Latinx educate this group on side effects, vaccine safety, and drug interactions. Additionally, the low rates of adherence to providers’ recommendation among minority populations have been linked to issues of trust, education, beliefs, and social factors that need further interventional investigations [40–42].

There was also a decreased likelihood of flu vaccination among those living alone, not covered by Medicare, or experiencing higher levels of financial strain. By reviewing the most recent available California Health Interview Survey (CHIS) pooled data (2014-2016) of South LA service area Latinx residents, 55 years of age and older, we determined that participants in our sample were almost 40% less likely to get the vaccine compared to the larger South LA 55+ population (73%) [43]. These findings suggest that our population differed in some very significant way from the overall Latinx population of South LA. It is possible that those reporting financial strain provides insight into why our sample may have differed from the pooled CHIS sample. It is also possible that the choice of recruitment sites (senior centers, senior living centers) may reflect some aspect of this population that bears deeper investigation.

When unpacking the concept of *financial strain*in this study, it is notable that the assessment questions reflect both necessities of life (food, clothing, and living expenses) as well as shared commodities in communal Latinx culture. A landmark review of familism by Baca Zinn (1983) perhaps best summarized this as “a positive form of social organization…” that facilitates adaptation to “marginal existence” associated with educational and economic disparities among Latinx [44]. In this group, periods of shortage impacting these necessities may be addressed through multigenerational living, allowing for communal access according to need [45]. Given that both living alone and financial strain were associated with not having flu vaccine in our sample, it is possible that some participants were unable to avail themselves of the physical, social and financial benefits of shared living [46, 47] It may also highlight communalistic, pro-social behaviors (such as willingness of a family member to provide transportation for medical appointments) that may act as vaccine facilitators in this group [48, 49].

Despite the potential benefit of shared living in terms of vaccine access, there have been recent data suggesting it may increase risk of contracting COVID-19 among Latinx [48]. Rising costs in the Southern California housing market and rising unemployment driven by the COVID-19 pandemic suggest that such larger household sizes may be a persistent trend and worthy of further exploration in terms of messaging around flu vaccination [50, 51].Additionally, interventions in senior centers and senior housing centers may address the need for flu vaccine promotion or delivery among aging Latinx who lack access to the extended family ties or the social support conferred by shared living [52].

Individuals with chronic medical conditions are considered high risk for influenza related complications, hospitalization and death [53]. While those participants in our sample not diagnosed with COPD or hypertension were less likely to report having a influenza vaccine within the previous 12 months, one striking finding of our study is the lack of a clear association between vaccination and a diabetes mellitus diagnosis. Like chronic respiratory and cardiovascular illness, the management of diabetes mellitus is benefited by regular medical appointments and monitoring every three to six months [54]. These frequent interactions with the healthcare system would result in a greater number of opportunities for providers to encourage or deliver impactful and cost-effective flu vaccinations [55]. Therefore, it is unclear why a diagnosis of COPD and/or hypertension might be protective in terms of flu vaccine, but diabetes mellitus, asthma and other chronic conditions examined in this study are not. As diabetes mellitus and coronary heart disease are two of the top five causes of death for Latinx persons in LA County, and two of the top five causes of premature death among residents of South LA, further exploration is needed to understand the mechanism behind these results [56].

Not having Medicare coverage was associated with a lower likelihood of having had a flu vaccine within the 12 months prior to the study. When unpacking the *Medicare coverage* variable in this population, immigration status is worthy of note and reflects larger phenomena impacting how Latinx access healthcare. Though it bears mentioning that it may also reflect the participant cohort (aged 55-65) not yet qualified to receive this coverage due to chronologic age.

This study connects difficulty accessing care and dissatisfaction with care delivery to a lack of influenza vaccination in the prior 12 months. A paucity of Latinx providers, with whom patients may identify, or who share language concordance, has been recognized as a persistent and growing problem in the US [55]. This dearth of culturally similar or informed providers can have an impact on both access and the quality of care [57–60]. For example, low-income, Latinx patients display a preference for an emotional connection with their providers [61, 62]. Studies in the safety net serving this demographic group have connected improved chronic illness management, patient disclosure and treatment adherence with displays of provider empathy and trust building [51–53]. Known barriers to patient-provider relationship building among Latinx (such as absence of “desahogarse” or the ability to unburdening oneself to a trusted person, or “respeto” the development of mutual respect) may have a similar, negative impact on flu vaccine uptake [51]. Recent work connecting provider burnout in the safety net to the quality and cultural acceptability of care delivery among aging Latinx populations may provide some guidance on how these challenges may be met [63, 64].

Study implications at the local level include a recognition of shifting demographics that may directly impact vaccine access for these ethnic minority elders. While African Americans have historically been the population majority in South LA, recent waves of immigration from Latin America have transformed the demographic make-up of this area [65]. Latinx now comprise the predominant racial/ethnic group in South LA [66], and social services traditionally focused on assisting historically under-resourced African American residents are struggling to keep up with this transition. Improving and adapting community outreach efforts in this area, especially in shared public spaces such as senior and community centers, as well as churches, may allow for better vaccine awareness and uptake among aging Latinx. Given the low number of participants in our sample reporting flu vaccination within the 12 months prior to this study, it appears that this culturally adapted messaging has yet to permeate these environments.

Targeted explorations that leverage existing, positive relationships in the South LA Latinx community might accelerate these efforts. The development of Latinx vaccine advocates (similar to promotoras or community health workers) might serve as conduits between the community and health care providers. The positive effects previously realized under such lay provider models might be similarly effective in promoting influenza vaccine uptake and addressing vaccine hesitancy [67–69]. These same efforts may increase COVID-19 vaccination rates.

Finally, this study shed light on a well-known issue facing the delivery of healthcare among racial and ethnic minority groups, namely the need for culturally tailored approaches and messaging. Influenza vaccine recommendations from providers were positively correlated with vaccination, indicating that Latinx elders found the delivery of this information quite helpful. However, knowing that 15% of participants indicated their providers never made this recommendation, and that almost one third of those who did receive it did not adhere, strongly suggests the need for further qualitative exploration to understand the mode(s) of failure. Provider training at the organizational or health system level addressing these issues might forestall miscommunication between providers and Latinx elders and have the added benefit of improving patient-provider relationships conducive to quality care delivery.

Limitations of this study include the use of consecutive sampling, which may inhibit generalizability of findings. Additionally, the vaccination rate within the 12 months prior to data collection in our sample was significantly different than the overall population of Latinx South LA residents, 55 years of age and older. However, we feel it represents data on barriers and facilitators of influenza vaccination among a difficult-to-reach population, within a population, of underserved, racial/ethnic minority elders. The nature of this cross-sectional study hampered our ability to make causal inferences, as did our inability to obtain detailed medical histories from our participants. This additional background information may have provided more robust insight on their influenza vaccine uptake practices.

Latinx elders who are not U.S. citizens may qualify for premium-free Medicare Part A benefits if they meet certain conditions, such as qualifying for Social Security retirement benefits, Social Security Disability (SSDI) or Railroad Retirement Benefits (RRB) [70]. While most people are automatically enrolled in Medicare at age 65, an additional application process may be required for these individuals. However, the process of applying for Medicare in person or online (even when qualified) may present a challenge to those who are hesitant due to fears of deportation or the impact it may have on subsequent application for citizenship [71]. Regulations promulgated in August 2019, the US Citizenship and Immigration Services (USCIS) “Public Charge Rule”, connected accessing social welfare programs to an inability to gain citizenship [61]. Wide-spread anti-immigrant rhetoric in the media during this period resulted in health behavior changes among US Latinx [72–75]. In LA County, it is most recently connected to a fear of accessing COVID-19 testing among undocumented Latinx [76]. The data used for this study were collected between the period of the “Public Charge Rule” proposal and its announced start date (February 2020). It is unclear how this may have hampered use of Medicare coverage for preventive services including flu vaccine.

## Conclusion

Influenza vaccine disparities among aging Latinx are well documented and encompass a variety of barriers reflecting the influence of culture, organizational and structural inequities built into our existing health systems and a legacy of unequal access to preventive care. Data from this study provides timely insight on these barriers, and others, that may both improve influenza vaccine uptake and provide critical insight into COVID-19 vaccine dissemination in a population disproportionately shouldering the human and financial costs of the coronavirus pandemic.

## Data Availability

The datasets used and/or analyzed during the current study are available from the corresponding author on reasonable request.

## References

[1] Rodriguez-Diaz CE, Guilamo-Ramos V, Mena L (2020). Risk for COVID-19 infection and death among Latinos in the United States: examining heterogeneity in transmission dynamics. Ann Epidemiol.

[2] Hooper MW, Nápoles AM, Pérez-Stable EJ. COVID-19 and racial/ethnic disparities. Jama. 2020. 10.1001/jama.2020.8598 PMC9310097 32391864

[3] Center KE, Da Silva J, Hernandez AL (2020). Multidisciplinary Community-Based Investigation of a COVID-19 Outbreak Among Marshallese and Hispanic/Latino Communities—Benton and Washington Counties, Arkansas, March–June 2020. Morb Mortal Wkly Rep.

[4] Podewils LJ, Burket TL, Mettenbrink C (2020). Disproportionate Incidence of COVID-19 Infection, Hospitalizations, and Deaths Among Persons Identifying as Hispanic or Latino—Denver, Colorado March–October 2020. Morb Mortal Wkly Rep.

[5] Riley AR, Chen Y-H, Matthay EC, et al. Excess deaths among Latino people in California during the COVID-19 pandemic. medRxiv. 2020. 10.1016/j.ssmph.2021.100860 PMC8283318 34307826

[6] Sáenz R, Garcia MA (2020). The disproportionate impact of COVID-19 on older Latino mortality: The rapidly diminishing Latino paradox.

[7] Kwai I (2021). US Starts Vaccine Rollout as High-Risk Health Care Workers Go First.

[8] Yang MJ, Rooks BJ, Le TT (2021). Influenza Vaccination and Hospitalizations Among COVID-19 Infected Adults. J Am Board Fam Med.

[9] Green I, Ashkenazi S, Merzon E, Vinker S, Golan-Cohen A. The association of previous influenza vaccination and coronavirus disease-2019. Hum Vaccin Immunother 2021:1-7. 10.1080/21645515.2020.1852010 PMC8189123 33377817

[10] Atallah F, Minkoff H (2021). During the second wave of COVID-19, don’t forget about influenza: a call to action. BJOG.

[11] Ruiz JB, Bell RA (2021). Predictors of intention to vaccinate against COVID-19: Results of a nationwide survey. Vaccine.

[12] US Centers for Disease Control and Prevention (2020). Disease Burden of Influenza.

[13] Hall LL, Xu L, Mahmud SM, Puckrein GA, Thommes EW, Chit A (2020). A Map of Racial and Ethnic Disparities in Influenza Vaccine Uptake in the Medicare Fee-for-Service Program. Adv Ther.

[14] Tse SC, Wyatt LC, Trinh-Shevrin C, Kwon SC (2018). Racial/Ethnic Differences in Influenza and Pneumococcal Vaccination Rates Among Older Adults in New York City and Los Angeles and Orange Counties. Prev Chronic Dis.

[15] Nowak GJ, Cacciatore MA, Len-Ríos ME (2018). Understanding and increasing influenza vaccination acceptance: insights from a 2016 national survey of US adults. Int J Environ Res Public Health.

[16] US Centers for Disease Control and Prevention (2020). Influenza Vaccination Coverage - FluVaxView.

[17] Zimmerman RK, Lauderdale DS, Tan SM, Wagener DK (2010). Prevalence of high-risk indications for influenza vaccine varies by age, race, and income. Vaccine.

[18] Nowak G, Cacciatore M, Len-Ríos M (2018). Understanding and increasing influenza vaccination acceptance: insights from a 2016 national survey of US adults. Int J Environ Res Public Health.

[19] Prins W, Butcher E, Hall LL, Puckrein G, Rosof B (2017). Improving adult immunization equity: Where do the published research literature and existing resources lead?. Vaccine..

[20] Bustamante AV, Chen J, Rodriguez HP, Rizzo JA, Ortega AN (2010). Use of preventive care services among Latino subgroups. Am J Prev Med.

[21] Kang-Kim M, Betancourt JR, Ayanian JZ, Zaslavsky AM, Yucel RM, Weissman JS (2008). Access to care and use of preventive services by Hispanics: state-based variations from 1991 to 2004. Med Care.

[22] DuBard CA, Gizlice Z (2008). Language spoken and differences in health status, access to care, and receipt of preventive services among US Hispanics. Am J Public Health.

[23] Wallace SP, Gutiérrez VF, Castañeda X (2008). Access to preventive services for adults of Mexican origin. J Immigr Minor Health.

[24] Pérez-Escamilla R, Garcia J, Song D (2010). Health care access among Hispanic immigrants:¿ Alguien está escuchando?[Is anybody listening?]. NAPA bulletin..

[25] Burger AE, Reither EN, Hofmann ET, Mamelund S-E (2018). The influence of hispanic ethnicity and nativity status on 2009 H1N1 pandemic vaccination uptake in the United States. J Immigr Minor Health.

[26] Bethel JW, Waterman SH (2009). Knowledge, attitudes and practices regarding influenza prevention and control measures among hispanics in San Diego County-2006. Ethn dis.

[27] Double Jeopardy: COVID-19 and Behavioral Health Disparities for Black and Latino Communities in the U.S. [press release]. 2020.

[28] Hall LL, Xu L, Mahmud SM, Puckrein GA, Thommes W, Chit A (2020). A map of racial and ethnic disparities in influenza vaccine uptake in the Medicare fee-for-service program. Adv Ther.

[29] Padilla ME, Frietze G, Shenberger-Trujillo JM, Carrillo M, Loya AM (2020). Influenza and intentions to vaccinate in an underserved Hispanic population: the role of theoretically derived constructs. J Pharm Pract.

[30] Pearson WS, Zhao G, Ford ES (2011). An analysis of language as a barrier to receiving influenza vaccinations among an elderly Hispanic population in the United States. Adv Prev Med.

[31] Phippard AE, Kimura AC, Lopez K, Kriner P (2013). Understanding knowledge, attitudes, and behaviors related to influenza and the influenza vaccine in US–Mexico border communities. J Immigr Minor Health.

[32] Wooten KG, Wortley PM, Singleton JA, Euler GL (2012). Perceptions matter: beliefs about influenza vaccine and vaccination behavior among elderly white, black and Hispanic Americans. Vaccine.

[33] Yoo B-K, Kasajima M, Phelps CE, Fiscella K, Bennett NM, Szilagyi PG (2011). Influenza vaccine supply and racial/ethnic disparities in vaccination among the elderly. Am J Prev Med.

[34] Bethel JW, Waterman SH (2009). Knowledge, attitudes and practices regarding influenza prevention and control measures among Hispanics in San Diego County–2006. Ethn Dis.

[35] Los Angeles County Department of Public Health (2017). Key Indicators of Health by Service Planning Area.

[36] Kahn JR, Pearlin LI (2006). Financial strain over the life course and health among older adults. J Health Soc Behav.

[37] Kan T, Zhang J (2018). Factors influencing seasonal influenza vaccination behaviour among elderly people: a systematic review. Public Health.

[38] Zimmerman RK, Nowalk MP, Bardella IJ (2004). Physician and practice factors related to influenza vaccination among the elderly. Am J Prev Med.

[39] Winston CA, Wortley PM, Lees KA (2006). Factors associated with vaccination of medicare beneficiaries in five US communities: Results from the racial and ethnic adult disparities in immunization initiative survey, 2003. J Am Geriatr Soc.

[40] Daniels NA, Juarbe T, Rangel-Lugo M, Moreno-John G, Perez-Stable EJ (2004). Focus group interviews on racial and ethnic attitudes regarding adult vaccinations. J Nat Med Assoc.

[41] Quinn SC, Parmer J, Freimuth VS, Hilyard KM, Musa D, Kim KH (2013). Exploring communication, trust in government, and vaccination intention later in the 2009 H1N1 pandemic: results of a national survey. Biosecur Bioterror.

[42] Chen JY, Fox SA, Cantrell CH, Stockdale SE, Kagawa-Singer M (2007). Health disparities and prevention: racial/ethnic barriers to flu vaccinations. J Community Health.

[43] Ask CHIS tools (2016). UCLA Center for Health Policy Research.

[44] Zinn MB (1982). FAMILISM AMONG CHICANOS: A THEORETICAL REVIEW. Humboldt J SocRelations..

[45] Under One Roof: A Review of Research on Intergenerational Coresidence and Multigenerational Households in the United States - Keene - 2010 - Sociology Compass - Wiley Online Library. Sociology Compass. 2010.

[46] @pewresearch. Record 64 million Americans live in multigenerational households. 2021.

[47] Muenning P. Living with parents or grandparents increases social capital and survival: 2014 General Social Survey-National Death Index. In: Jiao B, ed2018. 10.1016/j.ssmph.2017.11.001 PMC5769098 29349275

[48] Extended Families Living Together Raise Risks For COVID-19 Transmission. 2021.

[49] Ethnic socialization, identity, and values associated with U.S. Latino/a young adults’ prosocial behaviors. - PsycNET. 2021. 10.1037/cdp0000280 30920248

[50] @khouriandrew. Southern California median home price up 15% in September, sales jump as well. 2020.

[51] @pewresearch. Unemployment rose higher in three months of COVID-19 than it did in two years of the Great Recession. 2021.

[52] Kim D, Baum CF, Ganz ML, Subramanian SV, Kawachi I (2011). The contextual effects of social capital on health: a cross-national instrumental variable analysis. Soc Sci Med.

[53] (CDC) CfDC (2013). Prevention and control of seasonal influenza with vaccines. Recommendations of the Advisory Committee on Immunization Practices-United States, 2013–2014. MMWR Recomm.

[54] @CDCgov. Your Diabetes Care Schedule | Living with Diabetes | Diabetes | CDC. 2020.

[55] Maciosek MV, Solberg LI (2006). Influenza Vaccination: Health Impact and Cost Effectiveness Among Adults Aged 50 to 64 and 65 and Older. Am J Prev Med.

[56] Health LACDoP. MORTALITY IN LOS ANGELES COUNTY 2013. In. Leading Causes of Death and Premature Death with Trends for 2004-20132013:11, 27.

[57] Jetty A, Jabbarpour Y, Pollack J, Huerto R, Woo S, Petterson S. Patient-Physician Racial Concordance Associated with Improved Healthcare Use and Lower Healthcare Expenditures in Minority Populations. J Racial Ethn Health Disparities. 2021. 10.1007/s40615-020-00930-4 33403653

[58] Ortega P (2018). Spanish Language Concordance in US Medical Care: A Multifaceted Challenge and Call to Action. Acad Med.

[59] Parker MM, Fernández A, Moffet HH, Grant RW, Torreblanca A, Karter AJ (2017). Association of Patient-Physician Language Concordance and Glycemic Control for Limited-English Proficiency Latinos With Type 2 Diabetes. JAMA Intern Med.

[60] Villalobos BT, Bridges AJ, Anastasia EA, Ojeda CA, Rodriguez JH, Gomez D (2016). Effects of language concordance and interpreter use on therapeutic alliance in Spanish-speaking integrated behavioral health care patients. Psychol Serv.

[61] Services UCaI. Public Charge Fact Sheet. https://www.uscis.gov/news/public-charge-fact-sheet. Published Updated 09/22/2020. Accessed.

[62] Martinez-Hollingsworth A. Simultaneous experiences of Type 2 Diabetes and symptoms of depression and/or anxiety among Latina women 60 and older. (Dissertation, UCLA).2019.

[63] Martinez-Hollingworth A, Hicks, M., Chu, L & Kim, L. The Burnout Dyad: How Patients and Providers Co-Construct the Clinical Care Experience. Western Institute of Nursing, 53rd Annual Communicating Nursing Research Conference; 2019; Portland, OR.

[64] Martinez-Hollingsworth A. Podium: Effects of provider burnout: Working with Latino/a/x patients with multi-realm illness Paper presented at: Latinx Mental Health Workshop Series, Tarzana Treatment Centers2019; Tarzana, CA.

[65] USC Program for Environmental & Regional Equity (2017). An Equity Profile of the Los Angeles Region.

[66] Los Angeles County ISD. Los Angeles County Population Estimates (July 1, 2018). (http://www.lapublichealth.org/epi/docs/2018-LAC-Populationpdf. 6/26/2019, Accessted Jan 2021).

[67] Islam N, Riley L, Wyatt L (2014). Protocol for the DREAM Project (Diabetes Research, Education, and Action for Minorities): a randomized trial of a community health worker intervention to improve diabetic management and control among Bangladeshi adults in NYC. BMC Public Health..

[68] Pérez-Escamilla R, Damio G, Chhabra J (2015). Impact of a community health workers-led structured program on blood glucose control among latinos with type 2 diabetes: the DIALBEST trial. Diabetes Care.

[69] Cheun ASA, Loomis J (2018). A Culturally Sensitive Approach to Cervical Cancer Prevention in the Latina Population Using the Promotora Model. Nurs Womens Health.

[70] MedicareIntervactive.org. Medicare eligibility for non-U.S. citizens. https://www.medicareinteractive.org/get-answers/medicare-basics/medicare-eligibility-overview/medicare-eligibility-for-non-u-s-citizens. Published 2021. Accessed.

[71] Faber A (2020). A Vessel for Discrimination: The Public Charge Standard of Inadmissibility and Deportation.

[72] Haq C, Hostetter I, Zavala L, Mayorga J (2020). Immigrant Health and Changes to the Public-Charge Rule: Family Physicians’ Response. Ann Fam Med.

[73] Zallman L, Finnegan KE, Himmelstein DU, Touw S, Woolhandler S (2019). Implications of Changing Public Charge Immigration Rules for Children Who Need Medical Care. JAMA Pediatr.

[74] Kritz F (2019). Proposed Changes to the Public Charge Rule Could Imperil Immigrants’ Health. Am J Nurs..

[75] Doshi M, Lopez WD, Mesa H (2020). Barriers & facilitators to healthcare and social services among undocumented Latino(a)/Latinx immigrant clients: Perspectives from frontline service providers in Southeast Michigan. PLoS One.

[76] New ‘Public Charge’ Rule, Fear Of Deportation Prevents Many Immigrants From Seeking Care Even When Sick. KHN Morning Briefing. April 15, 2020, 2020.

